# Minerals and Trace Elements in 990 Beverages and Their Contribution to Dietary Reference Values for German Consumers

**DOI:** 10.3390/nu14224899

**Published:** 2022-11-19

**Authors:** Sebastian Schaffer, Gerald Rimbach, David Pieper, Niklas Hommen, Alexandra Fischer, Marc Birringer, Ulrike Seidel

**Affiliations:** 1 Institute of Human Nutrition and Food Science, University of Kiel, 24118 Kiel, Germany; 2 Department of Nutritional, Food and Consumer Sciences, University of Applied Sciences Fulda, 36037 Fulda, Germany

**Keywords:** beverages, minerals, dietary reference value, Germany, trace elements

## Abstract

Beverages are an integral part of human nutrition, yet little is known about their contribution to daily intakes of minerals and trace elements in German consumers. Using inductively coupled plasma-mass spectrometry, we determined the concentration of five minerals and six trace elements in beverage samples (*n* = 990, assigned to different beverage groups) collected throughout Germany. For a calculation of their relative contribution to the mineral supply, available beverage consumption data was combined with our quantitative analysis to calculate the average contribution of beverage groups to meet the respective dietary reference values currently used in Germany, Austria and Switzerland (D-A-CH region). Based on their presence in beverages and their consumption, the top three minerals are phosphorous, calcium and magnesium, and they, therefore, may reasonably contribute to the reference values. Among the trace elements, beverages mostly contributed to the manganese supply, whereas at the same time, concentrations of iron, cobalt and copper were low across all tested groups. Our study provides an overview of the assumed mineral and trace element intake via beverages in Germany and may, thus, serve as a foundation for a mineral and trace element database of beverages that needs to be expanded in the future.

## 1. Introduction

Minerals (e.g., potassium, calcium, magnesium) and trace elements (e.g., iron, copper, selenium) are essential nutrients for most life on earth [1]. An adequate intake of minerals—ideally via the daily diet—is crucial for many important metabolic functions such as enzyme activity, oxygen transport and bone formation, and thus ultimately, human health. In contrast, states of mineral deficiency often cause distinct disease patterns, for instance, osteoporosis (calcium deficiency) or anemia (iron deficiency) [2]. Hence, beverage consumption, for instance, in the form of mineral water and hot drinks, may be a suitable preventive and, in the case of medicinal waters, therapeutic means to maintain human well-being by preventing the onset and progression of mineral-related disease, in addition to its obvious hydrating properties. In 1957, epidemiological evidence for the correlation between minerals in water and health was published in Japan [3]. Since then, the effects of many beverages on consumer health have been investigated. The topics of published studies cover a wide range, from minerals to secondary plant metabolites to energy contents and beyond [4,5,6]. However, prior to any mineral-related health conclusions that can be drawn, it is obviously necessary to assess individual nutrient concentrations present in beverages to better understand their potential contribution to the mineral intake of consumers. This is particularly important, also considering the recent significant changes in age structure (for instance, the higher percentage of elderly people) and dietary habits (for instance, the rapid rise of vegetarians and vegans) observed in the German population in the past decade, thus warranting a scientific re-evaluation of the dietary mineral and trace element intake from beverages. For this, many countries around the world have established dietary reference values (DRVs) to guide nutrition research, policies, education, and food labeling, as recommended by the EFSA [7]. As DRVs vary considerably from country to country and typically undergo regular revisions, we consulted for our work the D-A-CH reference values (25-year-old to <51-year-old adults; average value for men and women) jointly published in Germany, Austria, and Switzerland [8].

**Figure 1:**
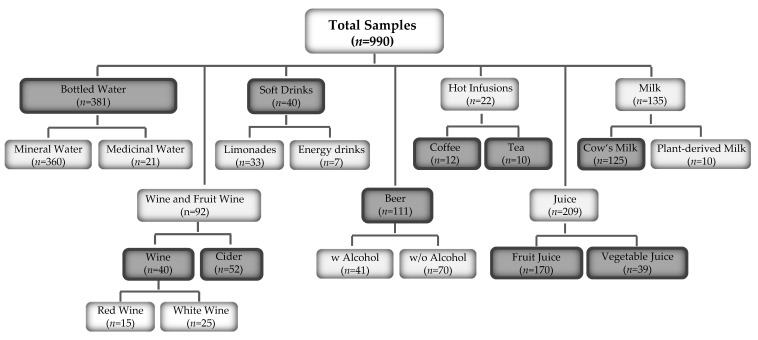
Overview of commercially available beverages in Germany that were included in this study (*n* = 990). Samples were divided into 7 main and 15 sub-groups. Groups for further analysis of per capita consumption are shown in dark grey.

In this study, we quantified the mineral and trace element concentration of selected minerals in 990 beverages belonging to 7 main groups (further divided into 15 sub-groups, Figure 1) collected throughout Germany. Among the minerals, we included potassium (K), sodium (Na), calcium (Ca), magnesium (Mg) and phosphorus (P) in our study. Among the trace elements, iron (Fe), zinc (Zn), copper (Cu), manganese (Mn), cobalt (Co) and selenium (Se) were quantified. With these data, we then calculated the percentage of minerals provided by coinciding beverage groups to meet the DRVs for adult consumers in Germany. Interestingly, despite being economically advanced and providing consumers with a wide range of food and beverage items, the undersupply of certain nutrients (e.g., of potassium, calcium, iodine) is still prevalent in some parts of the population. Hence, the knowledge obtained here is important to further improve not only our understanding of daily nutrient intake from beverages but also to better plan action programs in the field of public health nutrition.

## 2. Materials and Methods

### 2.1. Sampling of Beverages (Except Coffee and Tea)

Briefly, sampling took place between November 2017 and June 2021 throughout Germany. The sampling procedure and geographical distribution of mineral springs have already been described in detail in our previous publications [9,10]. Beverages were purchased from supermarkets, drinks retailers, petrol stations and kiosks. In total, 990 samples were collected (Figure 1). Ten-milliliter aliquots of the corresponding beverages were placed into polyethylene Falcon tubes with screw caps (Sarstedt, Nuembrecht, Germany). The samples were stored in carton boxes until mineral analysis.

### 2.2. Preparation of Coffee and Tea Samples

Coffee and tea samples were purchased in their dry form as 250–500 g ground coffee packages and commercially available tea bags, respectively. Coffee and tea infusions were prepared under standardized conditions: 7 g coffee ground was placed in a porcelain filter covered with unbleached filter paper and brewed with 150 mL deionized water at 90 °C. The tea bags (on average 2.1 g/bag; 1.75–3.0 g/bag) were brewed with 250 mL deionized water for 7 min at a starting temperature of 98 °C. Five-milliliter aliquots of the corresponding samples were dispensed into polyethylene Falcon tubes with screw caps (Sarstedt, Nuembrecht, Germany) and stored at −20 °C for multi-element analysis.

### 2.3. Analysis of Minerals and Statistical Analysis

Concentrations of minerals (potassium, sodium, calcium, magnesium and phosphorous) and trace elements (iron, zinc, copper, manganese, cobalt and selenium) were determined via an inductively coupled plasma-mass spectrometry (ICP-MS) ICAP Q instrument (Thermo Fisher Scientific, Waltham, MA, USA) at SGS Analytics (Jena, Germany). Measurements were conducted in accordance with DIN EN ISO 17294-2: 2017-01 and its respective safety requirements. Water samples were degassed and diluted 1 to 10 or 1 to 50 to reduce matrix effects. Beverage samples that possessed organic material were decomposed with a mixture of nitric acid and hydrogen peroxide (4:1) using microwave pressure digestion. For calibration, separate multi-element standards were used. To minimize spectroscopic interferences, a collision/reaction cell was used for detection. Rhodium (2 µg/L) was added as the internal standard. The ICP-MS analysis was fully validated with a ring trail of the German External Quality Assessment Scheme (G-EQUAS). Matrix-certified reference material of such ring trails was used for specific method validation.

**Figure 2:**
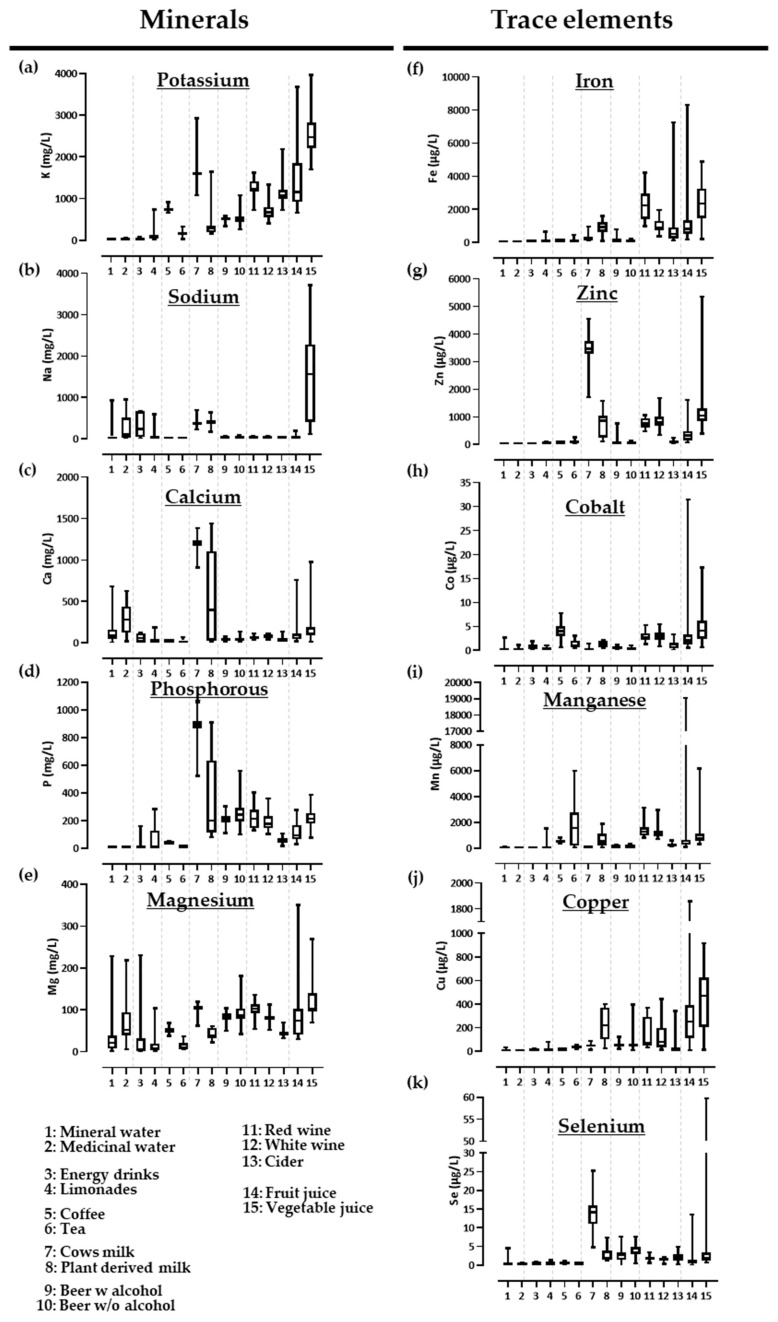
Mineral and trace element concentrations in commercially available beverages in Germany. (**a**–**e**) Mineral and (**f**–**k**) trace element concentrations (obtained via ICP-MS analyses) are shown as boxplots for 15 sub-groups of beverages.

Descriptive statistics and the preparation of corresponding graphs were conducted using the software GraphPad PRISM 9.3.1. The depicted boxplots in Figure 2 indicate median, minimum and maximum values and the 25% and 75% percentiles of mineral and trace element measurements. The calculation of the contribution of commercially available beverages in Germany to meeting the DRVs of minerals and trace elements was done with Microsoft Excel version 1808.

## 3. Results

### 3.1. Concentration of Minerals and Trace Elements in Commercially Available Beverages in Germany

To provide a visual summary enabling the identification of beverage groups of particular interest to mineral nutrient intake and, subsequently, to consumer health, the data for minerals and trace elements, resulting from the ICP-MS measurements, are given in the form of boxplots. As shown in Figure 2a–e, most beverage main and/or sub-groups contain small to moderate concentrations of minerals. Notable exceptions with high to very high concentrations (relative to the means, see Supplementary Table S1) were seen as follows: for potassium (Figure 2a), high concentrations were found in hot infusions of coffee (but not in tea), in cow’s milk and, to a lesser degree, in plant-based milk, wine and cider and in all juices, especially vegetable, citrus and exotic fruits juices. For sodium (Figure 2b), higher values were present in a few water and soft drink samples, cow’s and plant-derived milk and vegetable juices; for calcium (Figure 2c), in certain water samples, in cow’s milk and, to a lesser degree, plant-derived milk as well as in a few fruit and vegetable juices; and for phosphorous (Figure 2d), in cow’s milk and, to a lesser degree, in plant-derived milk, in a few fermented beverages, i.e., beer and wine and in a few fruit and vegetable juices. For magnesium (Figure 2e), all sample means were below or equal to about 100 mg/L; hence, a few water, soft drink and juice samples exhibited 2–3-fold higher concentrations.

As expected, trace element concentrations were, compared to minerals, generally lower by a factor of 10^3^ to 10^4^ (Figure 2f–k; Supplementary Table S1). As the mean and/or median values for the six analyzed trace elements were rather low (also in relation to their below-discussed potential to contribute to DRVs for adult consumers), we will focus hereinafter on a few notable exceptions seen in some beverage main and/or sub-groups. Moderate iron (Figure 2f) concentrations of about 1 mg/L were found in plant-derived milk and were, thus, 4fold higher than in cow’s milk, furthermore in red wine, with about 2 mg/L, and to a lesser degree in white wine and cider as well as in soft fruit juices, exotic fruit juices and vegetable juices (all > 1.5 mg/L). For zinc (Figure 2g), cow’s milk exhibited about 3.5 mg/L, four times the amount present in plant-derived milk, exotic fruit juices and vegetable juices. For manganese (Figure 2i), higher values were present in plant-derived milk, with about 0.7 mg/L, red and white wine, with more than 1 mg/L, soft fruit juices and vegetable juices, with about 1 mg/L, and exotic fruit juice, with about 7 mg/L. For copper (Figure 2j), the concentration in plant-derived milk was about 0.2 mg/L and in citrus-, soft-, exotic- and vegetable juices >0.35 mg/L. For selenium (Figure 2k), the concentration in cow’s milk was about 14 μg/L and in vegetable juices about 5 μg/L. All analyzed beverages did not appear to be sufficient sources of cobalt (values below 10 μg/L, Figure 2h).

### 3.2. Contribution of Commercially Available Beverages in Germany to Meet Dietary Reference Values (DRVs)

**Figure 3:**
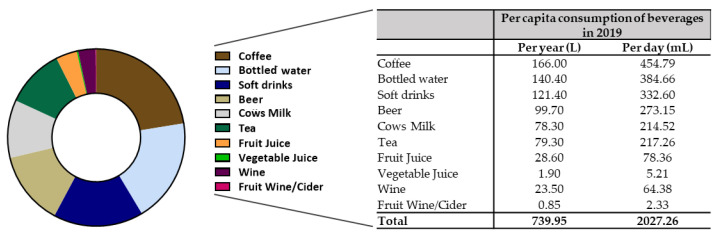
Published per capita consumption of beverages in Germany in 2019 [11,12,13]. Note: spirits, although depicted here, have not been further analyzed due to their low daily consumption.

Next, we calculated the contribution of commercially available beverages in Germany to the DRVs of the analyzed minerals and trace elements. For this, we first took the “per capita consumption” of beverages from published references [11,12,13] and subsequently calculated the daily intakes (Figure 3). In general, German consumers drink about 740 L of beverages per year, equivalent to a little more than 2 L per day. Thereby, coffee, bottled waters, soft drinks, beer and cow’s milk represent about 75% of the beverage intakes.

**Figure 4:**
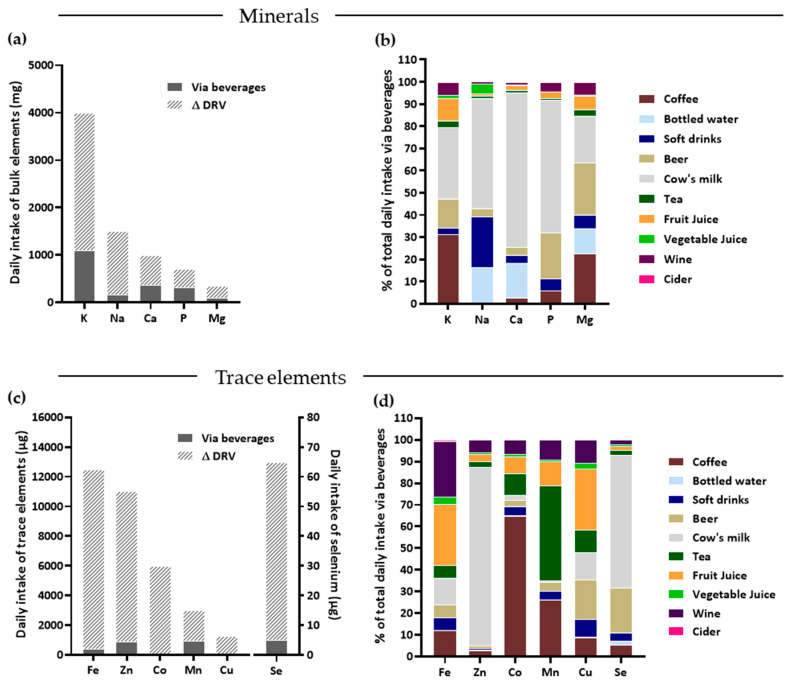
Contribution of commercially available beverages in Germany to meeting DRVs of selected minerals and trace elements. (**a**) Current DRVs (striped bars) in the D-A-CH region for five analyzed minerals as well the amount contributed via beverages (grey bars). (**b**) Contribution (in %) of ten beverage groups consumed in Germany to daily mineral intake. (**c**) Current DRVs (striped bars) in the D-A-CH region for six analyzed trace elements as well the amount contributed via beverages (grey bars). (**d**) Contribution (in %) of main beverage groups consumed in Germany to daily trace element intake. Beverage groups are based on those listed in Figure 3.

To evaluate the contribution to the DRVs, we used the data of minerals and trace elements from all beverage groups with available coinciding per capita consumption data (mean mineral/trace element concentration in beverage groups multiplied by the coinciding average daily beverage intake). As shown in Figure 4a, beverages have the potential to make substantial contributions to the DRVs for phosphorus (about 45%), calcium (about 37%), magnesium (about 29%) and potassium (about 27%). In contrast, beverages contribute rather little to sodium DRVs. Due to their relatively high daily consumption, the main contributors to mineral intakes are cow’s milk (potassium, sodium, calcium, phosphorous) and, to a lesser degree, coffee (potassium and magnesium), beer (phosphorous and magnesium) and bottled waters (sodium, calcium and magnesium) (Figure 4b). In contrast, beverages only contribute little to the DRVs of trace elements, except possibly for manganese (about 30%), zinc (about 8%) and selenium (about 8%) (Figure 4c). The main sources of trace elements in the beverages are cow’s milk (all except cobalt and manganese), coffee (cobalt, manganese and iron), tea (manganese, copper and cobalt), fruit juice (iron and copper), and beer (copper and selenium), as well as wine for the trace element iron (Figure 4d).

## 4. Discussion

### 4.1. Contribution of Beverages to Selected Mineral DRVs for German Consumers

Based on our quantitative analysis, beverages have the potential to provide noteworthy amounts to meet the DRVs of four minerals: calcium, phosphorous, potassium and magnesium.

#### 4.1.1. Calcium

Among all minerals in the human body, calcium is the most abundant, with a total mass of 1–2 kg in adults. Calcium levels in the human body are monitored by a complex system of calciotropic hormones [14]. Most nutritional societies recommend a calcium allowance between 700–1200 mg/day throughout life. In 2020, the German Nutrition Society (DGE) reported that more than 60 % of adult males and almost 80% of adult females did not meet the D-A-CH DRVs for daily calcium intake [15]. Of course, calcium metabolism is also affected by age, gender, genetics, the presence of other nutrients (e.g., oxalate, phosphate, sulfate, citrate, fiber and fats) and, most notably, sun exposure (vitamin D production) [16,17]. There is considerable controversy within the scientific community regarding the optimal amount of calcium intake, given the complex network of nutrient interactions. This discussion is further complicated by some studies highlighting a direct (though inconclusive) link between a high calcium intake (especially in the form of supplements) and an increased risk of cardiovascular diseases (CVDs) [18,19,20]. Based on our ICP-MS data and per capita beverage consumption, cow’s milk is the main liquid contributing to calcium intake via beverages. Of the 30% calcium DRV contributed by beverages, cow’s milk provides about 70%. Importantly, the calcium concentration in cow’s milk is about twice as high as that in plant-derived milk substitutes [21]. Despite the fortification of plant-based milk with different minerals and vitamins, a study in Switzerland analyzing 45 plant milk products found that replacing cow’s milk with plant milk led to a reduced intake of calcium, proteins, minerals, certain vitamins, and an increased intake of salt [22]. On the other hand, there is ongoing debate regarding the potential positive and negative health effects of cow’s milk in adults [21]. The fact that cow’s milk and plant-derived milk supply different nutrient profiles is of growing public health importance. During the last decade, cow’s milk consumption has declined in many parts of the world; at the same time, the intake of plant-derived milk substitutes grew worldwide at an annual growth rate of more than 10% and is estimated to reach a market volume of USD 24.6 billion in 2025 [23]. In the present study, the per capita consumption of plant milk was unfortunately not available for Germany but should be included in future evaluations.

Bottled water (mineral and medicinal water), on the other hand, was the second most important beverage supplying calcium to consumers, providing about 57 mg of calcium per day, equivalent to approximately 15% of the calcium DRV. However, it should be kept in mind that certain bottled waters (especially medicinal water products) contain much higher calcium concentrations, up to almost 700 mg/L, and, thus, may significantly contribute to the calcium DRVs in adults. These products may, thus, be of importance to those consumers avoiding dairy intake for health or environmental reasons [23]. Importantly, the bioavailability of calcium from bottled water is comparable to that from cow’s milk [24]. Of note, tap water consumption in Germany, which is around 70 mL per day [25] and is, hence, relatively low, was not considered in the current calculation.

#### 4.1.2. Phosphorous

The mineral phosphorus is intrinsically related to calcium homeostasis and function. Typical Western diets, with a high proportion of processed foods rich in additives (in the form of phosphoric acid, phosphates and polyphosphates), usually contain 1.5–2-fold of the recommended phosphorus intake of 700 mg. Consequently, phosphorus deficiencies are uncommon in these parts of the world [26,27]. As shown in Figure 4a, beverages provide about 40% of the phosphorous DRV to German consumers and, thus, likely contribute to the often-observed oversupply of this nutrient. The two main beverages with phosphorous are cow’s milk and beer. Concomitant to calcium, the phosphorous content is about 3 fold higher in cow’s milk compared to plant-derived milk substitutes. All other beverages analyzed in our study, including soft drinks, provided, on average, only negligible amounts of dietary phosphorus (Figure 4d), which is in accordance with data from the United States (3.3% of total phosphorus intake through soft drinks [27]). However, we found in some soft drinks phosphorus values of >200 mg/L. Hence, it should be kept in mind that excessive isolated intake of certain soft drinks, especially in children, could lead to high individual intakes of phosphorous, which is associated with altered bone metabolism, low bone density and fractures.

#### 4.1.3. Potassium

Potassium, an alkali metal, is the most abundant cation in intracellular fluids, and it participates in the regulation of excitable cells such as nerves and muscles [28]. Although present in most food items, especially fruits and vegetables, many Western populations do not fully meet the daily recommended potassium intake [29,30]. However, in 2017, an average potassium intake in the general German population of 3900 mg/day in women and 4300 mg/day in men was reported [31]. A low intake of potassium is of concern, considering more and more evidence suggesting a distinct role of potassium in maintaining healthy blood pressure or facilitating glucose tolerance [29,32]. In Germany, beverages contribute to approximately 20% of the potassium DRV, with cow’s milk and coffee as the main sources. Again, cow’s milk provides 4 fold more potassium compared to plant-derived milk substitutes, thus highlighting the rather high nutrient density of this animal-derived food. Among the juices, vegetable, citrus and exotic fruit juices have the potential to provide substantial amounts of potassium. Currently, German adults consume about 80 mL of fruit juice and 5 mL of vegetable juice daily, adding up to a total of about 85 mL. Assuming the entire volume would be consumed in the form of the three aforementioned juices, their relative contributions to meet the DRV would be 4.5% for vegetable juice and 3% for citrus and exotic fruit juices. Although this looks like a small addition to the daily potassium requirements, there have been intense efforts to increase fruit and vegetable consumption among German consumers [33]. Hence, replacing soft drinks (330 mL/day), for instance, with one glass (200 mL) of fruit or vegetable juice could increase the intake of potassium to a certain extent and, thus, contribute to meeting the DRVs of this important mineral. According to the findings from the German National Health Interview and Examination Survey, a steady increase in juice consumption over time (men: +7.5%, women: +3.2%) has been observed in German consumers, which was especially distinct in young and higher-educated men but was, in general, still lower than in women [34]. At the same time, in young men, the highest increase was in the intake of soft drinks (+27.6%), which may be a point of concern.

#### 4.1.4. Sodium

Sodium, another alkali metal, is mainly (95%) found in the extracellular fluid but is also deposited in other tissues, such as bone, skin and muscle. As sodium salts (e.g., sodium chloride) are ubiquitous in Western diets, sodium deficiency is uncommon in healthy adult Europeans [35,36,37,38]. In contrast, compelling evidence suggests that high dietary sodium intake in the form of sodium chloride contributes to hypertension, especially in salt-sensitive people [36,38,39]. Generally, for the prevention of high blood pressure and associated CVD, adults are advised to limit salt intake to 1 tablespoon, equivalent to 5 g of salt or 2000 mg of sodium [39,40]. In 2016, the observed median daily sodium intakes of German women and men reached 3.4 and 4.0 g, respectively, thus exceeding the levels recommended by German and international organizations [41]. Our data indicate that beverages only marginally contribute to sodium intake in German consumers. Hence, the sodium contents of beverages at the current per capita consumption levels are not likely to significantly affect human health in a positive or negative way. Even cow’s milk, which shows the highest sodium concentration of all beverages, does not add more than 80 mg (or less than 5%) of the DRV for this nutrient.

#### 4.1.5. Magnesium

Magnesium, an alkali earth metal, is a cofactor in more than 300 enzyme systems that regulate numerous biochemical reactions in the body, including energy production, muscle and nerve function, blood glucose control and blood pressure regulation [42,43,44,45]. Nearly 2/3 of the body’s magnesium content is deposited in the bones, where about 1/3 of magnesium is found intracellularly. Current D-A-CH DRVs for magnesium are 300 mg/d (adult women) and 350 mg/d (adult men), respectively [8].

Although magnesium is present in many food items and beverages, recent data indicate that magnesium intakes in Western countries are often inadequate [46]. Aside from diet-related factors, lifestyle, certain medications, diseases and pathophysiological conditions may contribute to magnesium deficiency [44,47]. With respect to beverages as a source of mineral supply to the human body, the interaction of magnesium with calcium and potassium ions are of particular interest. There is some evidence that magnesium intake may affect calcium retention and vice versa; hence, calcium supplementation (often promoted for the prevention of osteoporosis) in the absence of adequate magnesium intake can exacerbate a magnesium deficiency [48,49,50]. That such a scenario might be real is supported by data showing a significantly faster increase in calcium intake between 1977 and 2012, when US calcium intakes increased at a rate 2–2.5 times that of magnesium intakes [50]. Although evidence is still scarce, a calcium-to-magnesium ratio of 2.0 to <2.8 is generally considered critical for human health [50]. In our study, beverages contribute to about 1/3 of the magnesium DRV. The three biggest contributors, i.e., beer, cow’s milk, and coffee, all add about 5–7% of the DRV for this nutrient. These results are in good agreement with published data. Olechno et al. described the relative contribution of coffee as “an important source of magnesium, considering the risk of magnesium deficiency in modern societies” [51]. However, considering the potential health risks associated with the increased consumption of these beverages (e.g., high energy, in the case of cow’s milk; alcohol, in the case of beer; palpitation and/or insomnia, in the case of coffee), it seems counterproductive to recommend any beverage included in our study beyond a normal (moderate) intake level to improve the above-mentioned, often low magnesium status found in many consumers [51,52,53,54]. However, some medicinal and/or mineral waters exhibited >100–200 mg/L of magnesium by concomitant lower calcium levels in our study. Hence, their augmented consumption could contribute to a better magnesium supply. In fact, a significant increase in water consumption of +28% over time was observed in Germany [34], which was evident in men and women and in all age groups. Older people had the highest intake at any time, which could imply a contribution of water to the magnesium supply in this specific age group.

### 4.2. Contribution of Beverages to Selected Trace Element DRVs for German Consumers

Among the trace elements, our data showed that beverages contributed mostly to manganese intake, followed by zinc and selenium. In contrast, concentrations of iron, cobalt and copper were very low across all tested beverage main groups. Additionally, in cases where the latter three nutrients are present at higher concentrations (e.g., about 2 and 4 mg/L of iron in wine and soft fruit juices, respectively), the low daily consumption of these beverages does not supply a substantial percentage to the DRVs of these minerals.

#### 4.2.1. Manganese

In 2019, Sachse et al. reported an average dietary manganese intake in the general German population (age of 14–80 years) of about 2.8 mg/day for a person of 70 kg body weight. This intake level is within the DRV range of 2–5 mg/day recommended by the D-A-CH societies [55]. In agreement with previous studies, the two hot infusions (i.e., coffee and tea) are the two main contributors of manganese from beverages. However, direct comparisons with published data are difficult as the amount of manganese (and other minerals and trace elements) depends on various factors, such as water quality (deionized vs. normal water), brewing length, water temperature, and the origin of the tea (soil quality, altitude, etc.), to name a few [56]. Taken together, beverages—which supply about 30% of manganese DRVs in our study—are important sources of this trace element, and there appears to be little danger of manganese oversupply via beverages.

#### 4.2.2. Zinc

Zinc, the second most abundant trace element (after iron), is required for enzymatic, structural and signaling functions and is, thus, essential for all living organisms. In 2019, revised D-A-CH recommendations for zinc were published, with 7, 8 and 10 mg/d for adult women and 11, 14 and 16 mg/d for adult men, depending on low, medium, or high dietary phytate intakes, respectively [57]. Although nutrition societies stress that a balanced diet can supply adequate zinc to adult human consumers, they also acknowledge the presence of zinc deficiency in some fractions of society [57,58,59]. Of note, zinc bioavailability depends heavily on the presence of phytate from plant foods in the diet, suggesting that the currently ongoing shift to vegetarian and vegan diets observed worldwide might affect zinc supply over time. Among all tested beverages, only cow’s milk provided noteworthy quantities of zinc relative to its DRV. Importantly, zinc from animal sources exhibits a relatively high bioavailability due to the lack of phytate and other inhibitors. The presence of certain ligands, such as citrate or the sulfur-containing amino acid methionine, can further improve the bioavailability of zinc [60]. Based on our results, cow’s milk at current consumption levels provides about 6.8% of zinc DRV, about 4fold more than plant-derived milk substitutes, assuming equivalent voluminal intake. All other beverages only marginally contribute to reaching the DRVs for this trace element.

#### 4.2.3. Selenium

First considered toxic, selenium’s essentiality has been assumed since the 1950s [61] and verified after the discovery of selenium as an essential constituent of cellular glutathione peroxidase [62,63]. Selenium exerts its biological functions through selenoproteins and is involved, among others, in redox processes and antioxidant defense, thyroid hormone metabolism and signaling pathways [64]. In our study, cow’s milk and beer are the two main beverages contributing to selenium intake, providing more than 80% of the selenium consumed through beverages. The selenium concentration of food items heavily depends on the content of the soil used for plant and animal growth [65]. Although selenium in cow’s milk is readily bioavailable, there have been considerable research efforts to further increase its selenium content (biofortification), for instance, by adding yeast (organic selenium) or selenium compounds (e.g., sodium selenate) to the ruminant’s feed [65,66]. In 2004, Knowles et al. published selenium values of 3–15 μg/100 g of whole milk obtained from ruminants’ grazing pastures [67]. Considering that German adults consume about 200 mL of cow’s milk per day, this would translate to 6–30 μg of selenium per day (equivalent to approximately 10 to 50% of the DRV). However, we calculated only a daily selenium intake from cow’s milk of about 2–3 μg (less than 5% of selenium DRV), possibly suggesting that the selenium content of ruminants’ feed is relatively low. In fact, soils in Germany and many other parts of the world are rather low in selenium, thus providing a possible explanation for the observed low selenium concentration in cow’s milk and other food products [68,69]. Likewise, beer contributed to less than 1% of selenium DRV, which is in agreement with a study of selenium concentration in European beers [70]. Consequently, German consumers should take care to include more selenium-rich foods in their daily diet, especially meats, eggs, fish, nuts and mushrooms, to meet their selenium DRV [68].

### 4.3. Beverage Consumption in Germany in Comparison to Other Selected Countries

Our data indicate that due to either higher mineral content and/or higher consumption, some beverages, such as cow’s milk, bottled water or coffee, contribute more to the DRVs in Germany than others. Importantly in comparison to other countries, there are substantial differences in the consumption of beverages and, consequently, in their contribution in terms of mineral and trace element supply. Sigh et al. [71] conducted a systematic assessment of beverage intake (cow’s milk, water and soft drinks) in 187 countries around the world. They found significant heterogeneity in the consumption of each beverage by region (almost a 10-fold difference between the highest and the lowest) and age. The authors reported the highest intake of soft drinks in the Caribbean. Fruit juice consumption was highest in Australia and New Zealand, whereas Central Latin America and parts of Europe (Finland, Sweden) exhibited the highest milk intake. Intakes of all three beverages were lowest in East Asia and Oceania. Globally and within regions, soft drinks consumption was highest in younger adults, fruit juice consumption showed little relation with age, and milk intakes were highest in older adults. Data from the National Health and Nutrition Examination Survey of the United States revealed that water (tap and bottled) accounted for 51.2% of total non-alcoholic beverage consumption, followed by coffee, soft drinks, tea, fruit juice and cow’s milk. [72]. This is quite comparable with the consumption in Germany (Figure 3); hence, cow’s milk consumption was higher in Germany than in the US, and tap water was not taken into account in terms of German consumers.

Comparing three European countries, on average, the total water consumption was highest in France, followed by Spain, and lowest in Italy [73]. Only women in France, with 2.1 L of total water intake, consumed sufficient amounts according to EFSA reference values. Furthermore, beer consumption was relatively high in Germany (second highest among 28 evaluated European countries after the Czech Republic), whereas wine consumption was highest in France (Germany ranks in the middle) [74]. Comparing the percentage of caffeine-containing beverage volume sales, European countries, including Germany as well as North- and Latin American countries, had a relatively high intake of coffee, whereas in other Asian and African countries, tea was the most favorable beverage [75]. Consequently, the substantial differences in beverage consumption between countries and, hence, the different contributions to mineral and trace element supply in these countries should be considered and addressed in future studies.

### 4.4. Study Strengths and Limitations

Our study has several advantages, especially the utilization of state-of-the-art analytical technologies (ICP-MS) for the quantification of mineral and trace element concentrations. Furthermore, a hitherto not-yet-carried-out, relatively large number of beverages from several different groups was collected throughout Germany, thus providing a first fingerprint approximation of most mineral and trace element intakes via beverages in this geographical region. For some beverage groups (e.g., water, milk, juice), we identified a wide range in the mineral and trace element content among individual beverages. At the same time, it needs to be kept in mind that regional differences could not be taken into account and that some beverage groups were considerably larger than others, thus making exact comparisons between beverage groups and sub-groups, at times, difficult.

Likewise, it was not possible to ascertain the exact consumption of individual beverages within one group. We also did not conduct any bioavailability studies, a limitation that should be adequately addressed in future studies. 

## 5. Conclusions

In conclusion, the current study provides an overview of quantitative mineral and trace element concentrations in the main beverage groups consumed throughout Germany. Furthermore, our data suggest that beverages at current consumption levels are more suitable to contribute to a significant proportion of mineral DRVs (especially potassium, calcium and phosphorous) compared to trace element DRVs. Finally, the results of this study may lay the foundation of a comprehensive mineral and trace element database for beverages that needs to be expanded in the future.

## Data Availability

The data presented in this study are available upon reasonable request from the corresponding author.

## Supplementary Materials

The following supporting information can be downloaded at: https://www.mdpi.com/article/10.3390/nu14224899/s1, Table S1: Mean concentrations of minerals and trace elements and their contribution to DRVs.
