# Supplementary material for: Minerals and Trace Elements in 990 Beverages and Their Contribution to Dietary Reference Values for German Consumers
Source: Nutrients. 2022 Nov 19;14(22):4899. doi: 10.3390/nu14224899 (PMC9697264; doi:10.3390/nu14224899)
Supplement: Supplementary file 1 [file nutrients-14-04899-s001.zip › nutrients-2008113-supplementary.pdf]

Supplementary Table S1: Mean concentrations of minerals and trace elements and their contribution to DRV.

Mean concentrations of bulk and trace elements												Per head consumption 2019	
	Na<br>*mg/L*	Mg<br>*mg/L*	K<br>*mg/L*	Ca<br>*mg/L*	P<br>*mg/L*	Mn<br>*µg/L*	Fe<br>*µg/L*	Co<br>*µg/L*	Cu<br>*µg/L*	Zn<br>*µg/L*	Se<br>*µg/L*	Per year (L)	Per day (ml)
Coffee	1,15	51,73	743,9	22,5	40,66	543,2	107,3	4,07	15,22	56,07	0,59	166	454,79
Bottled water	68,5	30,67	5,62	149,2	0,06	3,16	1,2	0,04	0,61	2,84	0,2	140,4	384,66
Soft drinks	113,2	19,5	93,76	39,81	51,19	112,1	70,97	0,35	20,4	24,07	0,59	121,4	332,6
Beer	23,59	88,46	525	44,39	243,1	141,1	86,18	0,30	53,7	37,87	3,68	99,7	273,15
Cow's milk	381,1	103,7	1623	1199	887,9	27,7	234,2	0,316	47,65	3478	13,9	78,3	214,52
Tea	8,86	13,63	157,1	18,84	9,4	1917	107,3	1,28	39,08	104,4	0,45	79,3	217,26
Fruit Juice	15,28	79,19	1409	95,74	114,9	1337	1449	2,97	288,4	387,1	1,24	28,6	78,36
Vegetable Juice	1432	126,6	2560	182	222,3	1203	2491	4,63	432,3	1399	5,34	1,9	5,21
Wine	22,7	92,1	982,9	76,0	212,4	1351	1624	2,94	132,8	816,3	1,7	23,5	64,38
Cider	11,78	44,16	1120	46,07	58,52	276,3	951,8	1,081	28,98	93,61	2,143	0,85	2,33
**SUM**	**2078**	**650**	**9220**	**1874**	**1840**	**6912**	**7122**	**18,0**	**1059**	**6399**	**29,8**	**739,95**	**2027,26**
**Daily intake and contribution to DRV (absolute)**													
Coffee	0,52	23,53	338,32	10,23	18,49	247,04	48,8	1,85	6,92	25,5	0,27		
Bottled water	26,35	11,8	2,16	57,39	0,02	1,22	0,46	0,02	0,23	1,09	0,08		
Soft drinks	37,65	6,49	31,18	13,24	17,03	37,28	23,6	0,12	6,78	8,01	0,19		
Beer	6,44	24,16	143,4	12,13	66,4	38,54	23,54	0,08	14,67	10,34	1		
Cow's milk	81,75	22,25	348,17	257,21	190,47	5,94	50,24	0,07	10,22	746,1	2,98		
Tea	1,92	2,96	34,13	4,09	2,04	416,49	23,31	0,28	8,49	22,68	0,1		
Fruit Juice	1,2	6,21	110,4	7,5	9	104,76	113,54	0,23	22,6	30,33	0,1		
Vegetable Juice	7,45	0,66	13,33	0,95	1,16	6,26	12,97	0,02	2,25	7,28	0,03		
Wine	1,46	5,93	63,28	4,89	13,67	86,98	104,53	0,19	8,55	52,55	0,11		
Cider	0,027	0,103	2,608	0,107	0,136	0,643	2,217	0,003	0,067	0,218	0,005		
**SUM**	**164,8**	**104,1**	**1087,0**	**367,7**	**318,4**	**945,2**	**403,2**	**2,86**	**80,8**	**904,1**	**4,86**		
**DRV**	**1500**	**300-350**	**4000**	**1000**	**700**	**2000-5000**	**10000-15000**	**5000-8000**	**1000-1500**	**7000-16000**	**60-70**		
DRV for calculation	**1500**	**350**	**4000**	**1000**	**700**	**3000**	**12500**	**6000**	**1250**	**11000**	**65,0**		
△ DRV absolute	**1335**	**246**	**2913**	**632**	**382**	**2055**	**12097**	**5997**	**1169**	**10096**	**60,1**		
**Daily contribution to DRV (relative in %)**													
Coffee	0,03	6,72	8,46	1,02	2,64	8,23	0,39	0,03	0,55	0,23	0,41		
Bottled water	1,76	3,37	0,05	5,74	0	0,04	0	0	0,02	0,010	0,12		
Soft drinks	2,51	1,85	0,78	1,32	2,43	1,24	0,19	0	0,54	0,073	0,3		
Beer	0,43	6,9	3,59	1,21	9,49	1,28	0,19	0	1,17	0,094	1,55		
Cow's milk	5,45	6,36	8,7	25,72	27,21	0,2	0,4	0	0,82	6,78	4,59		
Tea	0,13	0,85	0,85	0,41	0,29	13,88	0,19	0	0,68	0,21	0,15		
Fruit Juice	0,08	1,77	2,76	0,75	1,29	3,49	0,91	0	1,81	0,28	0,15		
Vegetable Juice	0,5	0,19	0,33	0,09	0,17	0,21	0,1	0	0,18	0,066	0,04		
Wine	0,097	1,69	1,58	0,489	1,95	2,90	0,836	0,003	0,684	0,48	0,166		
Cider	0,002	0,007	0,174	0,007	0,009	0,043	0,148	0,000	0,004	0,015	0,000		
**SUM**	**11,0**	**29,7**	**27,1**	**36,8**	**45,5**	**31,5**	**3,2**	**0,0**	**6,5**	**8,2**	**7,5**		
△ DRV in %	89,0	70,3	72,9	63,2	54,5	68,5	96,8	100,0	93,5	91,8	92,5		
**Percentage of total daily intake via different beverages categories**													
Coffee	0,32	22,60	31,12	2,78	5,81	26,14	12,10	64,65	8,57	2,82	5,47		
Bottled water	15,99	11,34	0,20	15,61	0,01	0,13	0,11	0,60	0,29	0,12	1,62		
Soft drinks	22,85	6,23	2,87	3,60	5,35	3,94	5,85	4,12	8,39	0,89	4,01		
Beer	3,91	23,22	13,19	3,30	20,85	4,08	5,84	2,90	18,16	1,14	20,67		
Cow's milk	49,61	21,37	32,03	69,94	59,82	0,63	12,46	2,37	12,65	82,52	61,34		
Tea	1,17	2,85	3,14	1,11	0,64	44,06	5,78	9,68	10,51	2,51	2,00		
Fruit Juice	0,73	5,96	10,16	2,04	2,83	11,08	28,16	8,13	27,98	3,35	2,00		
Vegetable Juice	4,52	0,63	1,23	0,26	0,36	0,66	3,22	0,84	2,79	0,81	0,57		
Wine	0,89	5,70	5,82	1,33	4,29	9,20	25,92	6,62	10,58	5,81	2,21		
Cider	0,02	0,10	0,24	0,03	0,04	0,07	0,55	0,09	0,08	0,02	0,10		
**SUM (Control)**	**100**	**100**	**100**	**100**	**100**	**100**	**100**	**100**	**100**	**100**	**100**		
